# Low-Power Ultrasounds as a Tool to Culture Human Osteoblasts inside Cancellous Hydroxyapatite

**DOI:** 10.1155/2010/456240

**Published:** 2010-03-31

**Authors:** Lorenzo Fassina, Enrica Saino, Maria Gabriella Cusella De Angelis, Giovanni Magenes, Francesco Benazzo, Livia Visai

**Affiliations:** ^1^Dipartimento di Informatica e Sistemistica, University of Pavia, 27100 Pavia, Italy; ^2^Centre for Tissue Engineering (C.I.T.), University of Pavia, 27100 Pavia, Italy; ^3^Dipartimento di Biochimica, University of Pavia, 27100 Pavia, Italy; ^4^Dipartimento di Medicina Sperimentale, University of Pavia, 27100 Pavia, Italy; ^5^Dipartimento SMEC, IRCCS San Matteo, University of Pavia, 27100 Pavia, Italy

## Abstract

Bone graft substitutes and cancellous biomaterials have been widely used to heal critical-size long bone defects due to trauma, tumor resection, and tissue degeneration. In particular, porous hydroxyapatite is widely used in reconstructive bone surgery owing to its biocompatibility. In addition, the in vitro modification of cancellous hydroxyapatite with osteogenic signals enhances the tissue regeneration in vivo, suggesting that the biomaterial modification could play an important role in tissue engineering. In this study, we have followed a tissue-engineering strategy where ultrasonically stimulated SAOS-2 human osteoblasts proliferated and built their extracellular matrix inside a porous hydroxyapatite scaffold. The ultrasonic stimulus had the following parameters: average power equal to 149 mW and frequency of 1.5 MHz. In comparison with control conditions, the ultrasonic stimulus increased the cell proliferation and the surface coating with bone proteins (decorin, osteocalcin, osteopontin, type-I collagen, and type-III collagen). The mechanical stimulus aimed at obtaining a better modification of the biomaterial internal surface in terms of cell colonization and coating with bone matrix. The modified biomaterial could be used, in clinical applications, as an implant for bone repair.

## 1. Introduction

One of the key challenges in reconstructive bone surgery is to provide living constructs that possess the ability to integrate in the surrounding tissue. Bone graft substitutes, such as autografts, allografts, xenografts, and porous biomaterials have been widely used to heal critical-size long bone defects due to trauma, tumor resection, and tissue degeneration. The biomaterials used to build 3D scaffolds for bone tissue engineering are, for instance, the hydroxyapatite [1], the partially demineralized bone [2], biodegradable porous polymer-ceramic matrices [3], and bioactive glasses [4, 5]. 

The preceding osteoinductive and osteoconductive biomaterials are ideal in order to follow a typical approach of the tissue engineering, an approach that involves the seeding and the in vitro culturing of cells within a cancellous scaffold before the implantation.

The tissue-engineering method is of great importance. In order to overcome the drawbacks associated with the standard culture systems in vitro, such as limited diffusion and inhomogeneous cell-matrix distribution, several bioreactors have been designed to provide different physical stimuli: a rotating vessel bioreactor [6], a perfusion bioreactor [7], or an electromagnetic bioreactor [8], for instance. The ideal feature of a bioreactor is the supplying of suitable levels of oxygen, nutrients, cytokines, growth factors, and appropriate physical stimuli, in order to populate, with living bone cells and mineralized extracellular matrix, the volume of a porous biomaterial for reconstructive bone surgery: this living and biocompatible tissue-engineering construct could be implanted together with the insertion of a vascular pedicle [9].

Gorna and Gogolewski [10, 11] have drawn attention to the ideal features of a bone graft substitute: it should be porous with interconnected pores of adequate size (at least 200 *μ*m) allowing for the ingrowth of capillaries and perivascular tissues; it should attract mesenchymal stem cells from the surrounding bone and promote their differentiation into osteoblasts; it should avoid shear forces at the interface between bone and bone graft substitute; it should be biodegradable.

In this study, following the preceding “golden rules” of Gorna and Gogolewski, we have elected porous hydroxyapatite [12–14] as cancellous bone graft substitute and, using an ultrasonic stimulation [15], we have attempted to populate it with extracellular matrix and osteoblasts, of which cell function can be ultrasonically modulated [15].

Hydroxyapatite is widely used in reconstructive bone surgery owing to its biocompatibility. The in vitro modification of porous hydroxyapatite, with osteogenic signals of the transforming growth factor-*β* superfamily and with bone morphogenetic proteins, enhances the tissue regeneration in vivo [16], suggesting that the modification of hydroxyapatite could play an important role in tissue engineering.

As consequence, aiming, in a future work, at accelerated and enhanced bone regeneration in vivo, in the present study of tissue engineering, we show a particular “biomimetic strategy” that consists in the in vitro modification of porous hydroxyapatite with proliferated osteoblasts and their extracellular matrix produced in situ. In other words, applying an ultrasonic wave [15], our aim was to enhance a bone cell culture inside cancellous hydroxyapatite, that is, to coat the hydroxyapatite internal surface with physiological and biocompatible cell-matrix layers. Using this approach, the in vitro cultured material could be theoretically used, in clinical applications, as an osteointegrable implant.

## 2. Materials and Methods

### 2.1. Hydroxyapatite Disks

**Figure 1:**
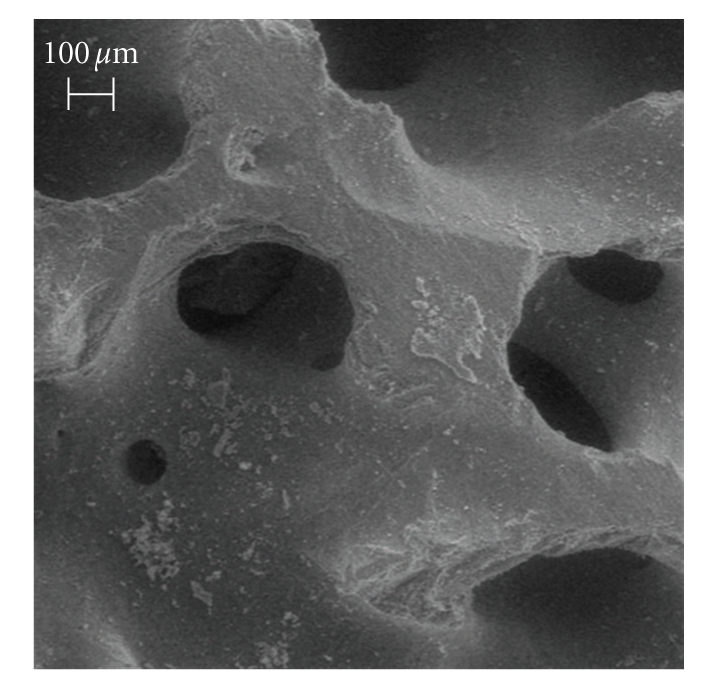
SEM image of unseeded hydroxyapatite, bar equal to 100 *μ*m.

Porous Orthoss bovine hydroxyapatite disks (diameter, 8 mm; height, 4 mm) were kindly provided by Geistlich Pharma AG (Wolhusen, Switzerland) [12–14]. The biomaterial had the following characteristics: internal surface area of 97 m^2^/g, average porosity equal to 60%, crystal dimensions of 10÷60 nm, and Ca/P ratio equal to 2.03, as in normal human cancellous bone (Figure 1).

### 2.2. Cells

The human osteosarcoma cell line SAOS-2 was obtained from the American Type Culture Collection (HTB85, ATCC, Rockville, MD). The cells were cultured in McCoy's 5A modified medium with l-glutamine and HEPES (Cambrex Bio Science Baltimore, Inc., Baltimore, MD), supplemented with 15% fetal bovine serum, 2% sodium pyruvate, 1% antibiotics, 10^−8^ M dexamethasone, and 10 mM *β*-glycerophosphate (Sigma-Aldrich, Inc., Milwaukee, WI). Ascorbic acid, another osteogenic supplement, is a component of McCoy's 5A modified medium. The cells were cultured at 37°C with 5%  CO_2_, routinely trypsinized after confluency, counted, and seeded onto the hydroxyapatite disks.

### 2.3. Cell Seeding

In order to anchor the hydroxyapatite disks to two standard well-plates, 3% (w/v) agarose solution was prepared and sterilized in autoclave, and during cooling, at 45°C, 100 *μ*L of agarose solution were poured inside the wells to hold the placed hydroxyapatite disks and to fix them after completed cooling.

The well-plates with the biomaterial disks were sterilized by ethylene oxide at 38°C for 8 hours at 65% relative humidity. After 24 hours of aeration in order to remove the residual ethylene oxide, the disks were ready inside the two culture systems: the “static,” that is, the control well-plate without external stimulus and the “ultrasonic,” that is, the ultrasonically stimulated well-plate.

A cell suspension of 10 × 10^6^ cells in 400 *μ*L was added onto the top of each disk and, after 0.5 hour, 600 *μ*L of culture medium was added to cover the disks. Cells were allowed to attach overnight, then the static culture was continued in the standard well-plate and the ultrasound stimulation was applied for the first time.

### 2.4. Ultrasound Stimulation

An ultrasound stimulus [15] was applied through the culture medium by a FAST ultrasound generator (Igea, Carpi, Italy) to the seeded hydroxyapatite disks. The mechanical wave had the following characteristics: signal frequency equal to 1.5 ± 0.03 MHz, duty cycle of 200 ± 4 *μ*s, repetition rate equal to 1 ± 0.02 kHz, and temporal average power of 149 ± 3 mW. Low-intensity ultrasound stimulus accelerates the fracture healing in clinical studies [17].

The ultrasonic culture was placed into a standard cell culture incubator with an environment of 37°C and 5% CO_2_, and it was stimulated 20 min/day for a total of 22 days. The culture medium was changed on days 4, 7, 10, 13, 16, and 19.

### 2.5. Standard Well-Plate Culture

The static culture was placed into a standard cell culture incubator. The duration of the static culture was 22 days and the culture medium was changed on days 4, 7, 10, 13, 16, and 19.

### 2.6. Scanning Electron Microscopy (SEM) Analysis

At the end of the culture period, the disks were fixed with 2.5% (v/v) glutaraldehyde solution in 0.1 M Na-cacodylate buffer (pH = 7.2) for 1 hour at 4°C, washed with Na-cacodylate buffer, and then dehydrated at room temperature in a gradient ethanol series up to 100%. The samples were kept in 100% ethanol for 15 minutes, and then critical point-dried with CO_2_. The specimens were mounted on aluminum stubs, sputter coated with gold (degree of purity equal to 99%), and then observed with a Leica Cambridge Stereoscan microscope (Leica Microsystems, Bensheim, Germany).

### 2.7. DNA Content

At the end of the culture period, the cells were lysed by a freeze-thaw method in sterile deionized distilled water and the released DNA content was evaluated with a fluorometric method (PicoGreen, Molecular Probes, Eugene, OR). A DNA standard curve [15], obtained from a known amount of osteoblasts, was used to express the results as cell number per disk.

### 2.8. Set of Rabbit Polyclonal Antisera

Fisher et al. (http://csdb.nidcr.nih.gov/csdb/antisera.htm, National Institutes of Health, National Institute of Dental and Craniofacial Research, Craniofacial and Skeletal Diseases Branch, Matrix Biochemistry Unit, Bethesda, MD) presented us, generously, with the following rabbit polyclonal antibody immunoglobulins G: antiosteocalcin, anti-type-I collagen, anti-type-III collagen, antidecorin, and antiosteopontin (antiserum LF-32, LF-67, LF-71, LF-136, and LF-166, respectively) [18].

### 2.9. Set of Purified Proteins

Decorin [19], osteocalcin (immunoenzymatic assay kit, BT-480, Biomedical Technologies, Inc., Stoughton, MA), osteopontin (immunoenzymatic assay kit, 900-27, Assay Designs, Inc., Ann Arbor, MI), type-I collagen [20], and type-III collagen (Sigma-Aldrich) were used.

### 2.10. Confocal Microscopy

At the end of the culture period, the disks were fixed with 4% (w/v) paraformaldehyde solution in 0.1 M phosphate buffer (pH = 7.4) for 8 hours at room temperature and washed with PBS (137 mM NaCl, 2.7 mM KCl, 4.3 mM Na_2_HPO_4_, 1.4 mM KH_2_PO_4_, pH = 7.4) three times for 15 minutes. The disks were then blocked by incubating with PAT (PBS containing 1% [w/v] bovine serum albumin and 0.02% [v/v] Tween 20) for 2 hours at room temperature and washed.

L. Fisher's antidecorin, antiosteocalcin, antiosteopontin, anti-type-I collagen, and anti-type-III collagen rabbit polyclonal antisera were used as primary antibodies with a dilution equal to 1 : 1000 in PAT. The incubation with the primary antibodies was performed overnight at 4°C, whereas the negative controls were based upon the incubation, overnight at 4°C, with PAT instead of the primary antibodies. The disks and the negative controls were washed and incubated with Alexa Fluor 488 goat antirabbit IgG (H+L) (Molecular Probes) with a dilution of 1 : 500 in PAT for 1 hour at room temperature. 

At the end of the incubation, the disks were washed in PBS, counterstained with Hoechst solution (2 *μ*g/mL) to target the cellular nuclei, and then washed. The images were taken by blue excitation with a confocal microscope (TCS SPII, Leica Microsystems) equipped with a digital image capture system at 100× magnification.

### 2.11. Extraction of the Extracellular Matrix Proteins from the Cultured Disks and Enzyme-Linked Immunosorbent Assay (ELISA)

At the end of the culture period, in order to evaluate the amount of the extracellular matrix constituents over the internal and external hydroxyapatite surfaces, the disks were washed extensively with sterile PBS (137 mM NaCl, 2.7 mM KCl, 4.3 mM Na_2_HPO_4_, 1.4 mM KH_2_PO_4_, pH = 7.4) in order to remove the culture medium, and then incubated for 24 hours at 37°C with 1 mL of sterile sample buffer (1.5 M Tris-HCl, 60% [w/v] sucrose, 0.8% [w/v] sodium dodecyl sulphate, pH = 8.0). At the end of the incubation period, the sample buffer aliquots were removed, and then the disks were centrifuged at 4000 rpm for 15 minutes in order to collect the sample buffer entrapped into the pores. The total protein concentration in the two culture systems was evaluated by the BCA Protein Assay Kit (Pierce Biotechnology, Inc., Rockford, IL). The total protein concentration was 749 ± 108 *μ*g/mL in the static culture and 1527 ± 274 *μ*g/mL in the ultrasonic culture (*P* < .05). After matrix extraction, the disks were incubated, once again, for 24 hours at 37°C with 1 mL of sterile sample buffer, and no protein content was detected.

Calibration curves to measure decorin, osteocalcin, osteopontin, type-I collagen, and type-III collagen were performed. Microtiter wells were coated with increasing concentrations of each purified protein, from 1 ng to 2 *μ*g, in coating buffer (50 mM Na_2_CO_3_, pH = 9.5) overnight at 4°C. Some of the wells were coated with bovine serum albumin (BSA) as a negative control. In order to measure the extracellular matrix amount of each protein by an ELISA, microtiter wells were coated, overnight at 4°C, with 100 *μ*L of the extracted extracellular matrix (20 *μ*g/mL in coating buffer). After three washes with PBST (PBS containing 0.1% [v/v] Tween 20), the wells were blocked by incubating with 200 *μ*L of PBS containing 2% (w/v) BSA for 2 hours at 22°C. The wells were subsequently incubated for 1.5 hours at 22°C with 100 *μ*L of the L. Fisher's antidecorin, antiosteocalcin, antiosteopontin, anti-type-I collagen, and anti-type-III collagen rabbit polyclonal antisera (1 : 500 dilution in 1% BSA). After washing, the wells were incubated for 1 hour at 22°C with 100 *μ*L of HRP-conjugated goat anti-rabbit IgG (1 : 1000 dilution in 1% BSA).

The wells were finally incubated with 100 *μ*L of development solution (phosphate-citrate buffer with *o*-phenylenediamine dihydrochloride substrate). The color reaction was stopped with 100 *μ*L of 0.5 M H_2_SO_4_ and the absorbance values were measured at 490 nm with a microplate reader (Bio-Rad Laboratories, Inc., Hercules, CA). The amount of extracellular matrix constituents inside the disks is expressed as fg/(cell × disk).

### 2.12. Statistics

The disks number was 24 in each repeated experiment (12 disks in the control culture and 12 disks in the ultrasonic culture). The experiment was repeated 4 times. Results are expressed as mean ± standard deviation. In order to compare the results between the two culture systems, one-way analysis of variance (ANOVA) with *post hoc* Bonferroni test was applied, electing a significance level of 0.05.

## 3. Results

The human SAOS-2 osteoblasts were seeded onto porous hydroxyapatite disks, and then cultured without or with an ultrasonic stimulus for 22 days. These culture methods permitted the study of the SAOS-2 cells as they modified the biomaterial through the proliferation and the coating with extracellular matrix. The cell-matrix distribution was compared between the two culture systems.

### 3.1. Microscope Analysis

**Figure 2:**
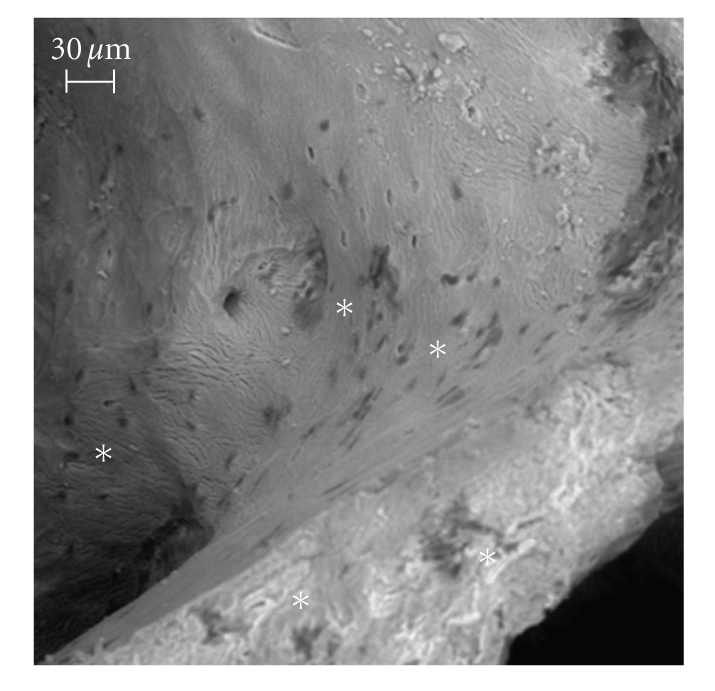
SEM image of the static culture, bar equal to 30 *μ*m. The osteoblasts are in the “backscattered depressions” near the juxtaposed asterisks: at the end of the culture period, statically cultured cells were few and, essentially, not surrounded by extracellular matrix; therefore, wide biomaterial regions remained devoid of cell-matrix complexes.

**Figure 3:**
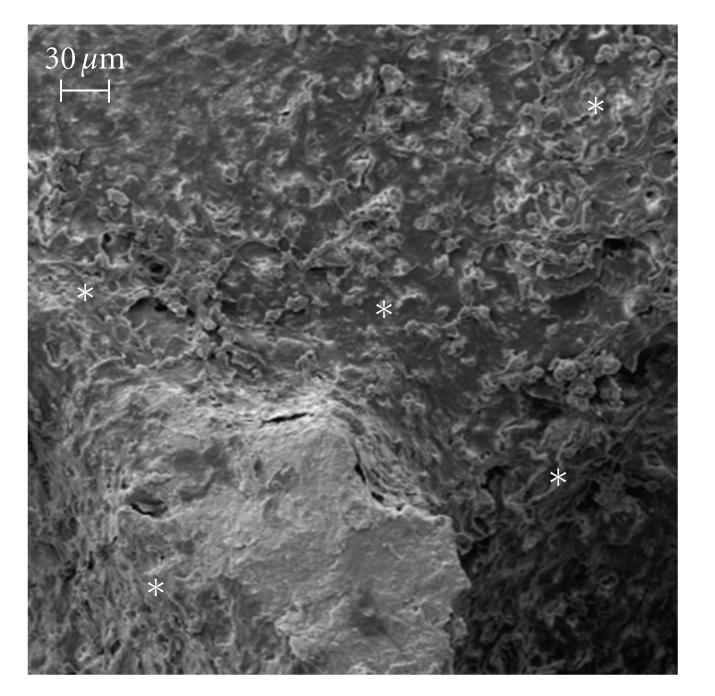
SEM image of the ultrasonic culture, bar equal to 30 *μ*m. During the culture period, the physical stimulus caused a wide-ranging coat of the internal surface of the biomaterial: several osteoblasts proliferated and the biomaterial was tending to be hidden by cell-matrix layers (asterisks).

In comparison to control condition, SEM images revealed that, due to the ultrasound stimulus, the osteoblasts proliferated and built their extracellular matrix over the available internal hydroxyapatite surface (Figures 2 and 3). At the end of the culture period, statically cultured cells were few and, essentially, not surrounded by extracellular matrix, therefore wide biomaterial regions remained devoid of cell-matrix complexes (Figure 2). In contrast, the physical stimulus caused a wide-ranging coat of the internal surface of the biomaterial: several osteoblasts proliferated and the biomaterial was tending to be hidden by cell-matrix layers (Figure 3).

**Figure 4:**
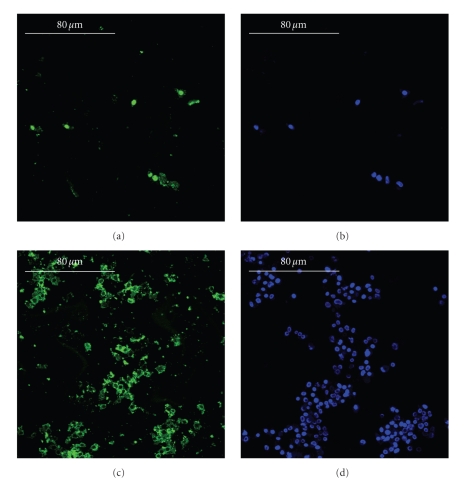
Immunolocalization of type-I collagen (panels a and c, green) and cellular nuclei (panels b and d, blue) in the static culture (panels a and b) and in the ultrasonic culture (panels c and d), bars equal to 80 *μ*m. During the culture period, in the control (panels a and b), the osteoblasts built a scanty amount of bone matrix, whereas, in the stimulated culture (panels c and d), the osteoblasts secreted a wide amount of matrix. The immunolocalization of osteocalcin, osteopontin, and type-III collagen revealed similar results.

**Figure 5:**
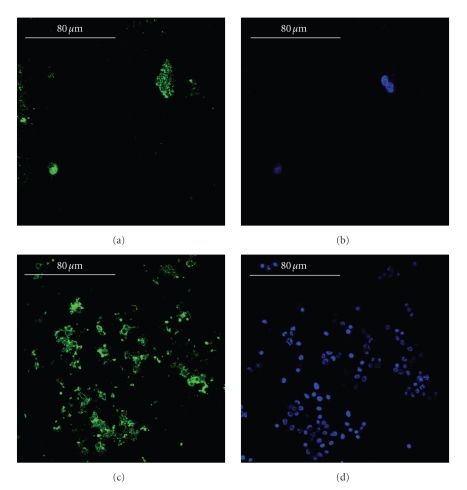
Immunolocalization of decorin (panels a and c, green) and cellular nuclei (panels b and d, blue) in the static culture (panels a and b) and in the ultrasonic culture (panels c and d), bars equal to 80 *μ*m. During the culture period, in the control (panels a and b), the osteoblasts produced a very little amount of decorin, a key regulator for matrix spatial organization, whereas, in the stimulated culture (panels c and d), the osteoblasts secreted a larger amount of 3D organized bone matrices.

The immunolocalization of type-I collagen and decorin with the counterstaining of the cellular nuclei showed the stimulation effects in terms of higher cell proliferation and more intense building of the extracellular matrix (Figures 4 and 5). The immunolocalization of osteocalcin, osteopontin, and type-III collagen revealed similar results (data not shown).

These observations were confirmed by the measure of the DNA content at the end of the culture period: in the static culture, the cell number per disk grew to 22.1 × 10^6^ ± 3.2 × 10^4^ and in the ultrasonic culture to 34.7 × 10^6^ ± 3.9 × 10^4^ with *P* < .05.

### 3.2. Extracellular Matrix Extraction

In order to evaluate the amount of bone extracellular matrix inside the hydroxyapatite disks, an ELISA of the extracted matrix was performed: at the end of the culture period, in comparison with the static culture, the ultrasound stimulation significantly increased the internal surface coating with decorin, osteocalcin, osteopontin, type-I collagen, and type-III collagen (*P* < .05) (Table 1).

## 4. Discussion

The aim of this study was the in vitro modification of a porous hydroxyapatite with extracellular matrix and osteoblasts to make the biomaterial more biocompatible for the bone repair in vivo.

A discussion about the concept of “biocompatibility” is necessary. When a biomaterial is implanted in a biological environment, a nonphysiologic layer of adsorbed proteins mediates the interaction of the surrounding host cells with the material surface. The body interprets this protein layer as a foreign invader that must be walled off in an avascular and tough collagen sac. Therefore, the biomedical surfaces must be developed so that the host tissue can recognize them as “self”. Castner and Ratner think the “biocompatible surfaces” of the “biomaterials that heal” as the surfaces with the characters of a “clean, fresh wound” [21]: these “self-surfaces” could obtain a physiological inflammatory reaction leading to normal healing. In this study, we have followed a biomimetic strategy where the seeded osteoblasts built a biocompatible surface made of bone matrix [15, 22].

To enhance the coating of the biomaterial internal surface, an ultrasonic wave was applied to the seeded biomaterial [15]. The ultrasound stimulus increased the cell proliferation around 1.6-fold. Furthermore, the ultrasonic wave significantly enhanced the synthesis of type-I collagen, decorin, osteopontin, osteocalcin, and type-III collagen, which are fundamental constituents of the physiological bone matrix: in particular, type-I collagen is the most important and abundant structural protein of the bone matrix; decorin is a proteoglycan considered a key regulator for the assembly and the function of many extracellular matrix proteins with a major role in the lateral growth of the collagen fibrils, delaying the lateral assembly on the surface of the fibrils; osteopontin is an extracellular glycosylated bone phosphoprotein secreted at the early stages of the osteogenesis before the onset of the mineralization, it binds calcium, it is likely to be involved in the regulation of the hydroxyapatite crystal growth, and, through specific interaction with the vitronectin receptor, it promotes the attachment of the cells to the matrix; osteocalcin is secreted after the onset of mineralization and it binds to bone minerals.

The preceding results could be explained with a signaling model. The ultrasound stimulation raises the net Ca^2+^ flux in the osteoblast cytosol and the release of the intracellular Ca^2+^ [23–25]. According to Pavalko's signaling model, the increase of the cytosolic Ca^2+^ concentration is the starting point of signaling pathways, which cause the secretion of prostaglandins enhancing the osteoblast proliferation, and which target specific bone matrix genes [23].

Consistent with Pavalko's model, mechanically stimulated osteoblasts produce autocrine and paracrine prostaglandin signal for cell proliferation; the same mechanically stimulated osteoblasts produce bone extracellular matrix. Prostaglandins are released in the culture medium, whereas the proteins are deposited onto the biomaterial. Even if prostaglandins and proteins have partially common biochemical pathways [23], they have a different geometrical destination: the medium and the material surface, respectively. For that reason, the efficiency in prostaglandin action (cell proliferation enhancement of 1.6-fold) was different from the efficiency of matrix deposition (biomaterial coating enhancement of 1.7÷4.5-fold as in Table 1).

In this study, the ultrasonic stimulus was a physical method to obtain the biomimetic modification of the material, whose internal surface was coated by osteoblasts and by a layer of bone matrix. The use of a cell line showed the potential of the ultrasound stimulation; nevertheless, appropriately tuning the parameters of the ultrasonic wave, the stimulus duration, and the culture time, a better result could be obtained with autologous bone marrow stromal cells instead of SAOS-2 osteoblasts for total immunocompatibility with the patient. In addition, after the in vivo implantation of the cultured cancellous hydroxyapatite, an ultrasound therapy could be applied with the same wave parameters [15] to enhance the patient healing [17].

In conclusion, we theorize that the cultured “self-surface” could be used fresh, that is, rich in autologous cells and matrix, or after sterilization with ethylene oxide, that is, rich only in autologous matrix. In future work, we intend to use our constructs, which are rich in autologous matrix, as a simple, storable, tissue-engineering product for the bone repair [22].

## Figures and Tables

**Table 1:** Amount of extracellular matrix constituents inside hydroxyapatite.

	Matrix protein total coating after 22 days of culture in fg/(cell × disk)		
	Static culture	Ultrasonic culture	Ultrasonic /Static
Decorin	5.58 ± 0.22	15.25 ± 0.42	2.73-fold
Osteocalcin	1.79 ± 0.33	5.76 ± 0.39	3.22-fold
Osteopontin	1.75 ± 0.73	3.04 ± 0.47	1.74-fold
Type-I collagen	3.72 ± 0.49	16.85 ± 0.95	4.53-fold
Type-III collagen	4.59 ± 0.13	11.04 ± 0.71	2.40-fold

Table note: *P* < .05 in all “Static” versus “Ultrasonic” comparisons.
